# Supplementary material for: Carbon defect qubit in two-dimensional WS2
Source: Nat Commun. 2022 Mar 8;13:1210. doi: 10.1038/s41467-022-28876-7 (PMC8904548; doi:10.1038/s41467-022-28876-7)
Supplement: Supplementary file 1 — Supplementary Information [file 41467_2022_28876_MOESM1_ESM.pdf]

Supplementary Information

Carbon defect qubit in two-dimensional WS_2

Song Li, Gergő Thiering, and Péter Udvarhelyi
Wigner Research Centre for Physics, P.O. Box 49, H-1525 Budapest, Hungary

Viktor Ivády
Wigner Research Centre for Physics, P.O. Box 49, H-1525 Budapest, Hungary
Department of Physics, Chemistry and Biology, Linköping University, 581 83 Linköping, Sweden and
Max Planck Institute for the Physics of Complex Systems, Nöthnitzer Straße 38, 01187 Dresden, Germany

Adam Gali
Wigner Research Centre for Physics, P.O. Box 49, H-1525 Budapest, Hungary and
Department of Atomic Physics, Institute of Physics,
Budapest University of Technology and Economics,
Műegyetem rakpart 3., H-1111 Budapest, Hungary

(Dated: February 9, 2022)

SUPPLEMENTARY NOTE 1: ASSESSMENT OF DENSITY FUNCTIONAL THEORY

The two-dimensional nature of layered transition metal dichalgonides (TMDCs) have large exciton binding energy. Experiment measures the exciton binding energy is about 0.37 eV [1] for WSe_2 and 0.32-0.71 eV in WS_2 [2, 3]. To accurately consider the exciton effect and calculate the fundamental band gap, GW approximation with Bethe-Salpeter equation (BSE) should be used. Previous studies indicate that the quasiparticle band gap of WS_2 is about $2.8 - 2.9$ eV and the exciton binding energy is about 0.6 eV [4–6]. However, GW calculation is extremely time consuming and it is usually out of reach for defective supercell systems. To reach a compromise, in this paper we slightly modify the parameters of DFT HSE06 functional through mixing parameter α and the range separation parameter μ to yield reasonable band gap [7, 8], as shown in Supplementary Figure 1. With $\alpha = 0.40$ and $\mu = 0.10$ Å^{-1}, the calculated bandgap is 2.76 eV with SOC included which is close to the experimental value. We call this functional HSE throughout the paper.

The SOC effect for the pristine WS_2 is evaluated in Supplementary Figure 2. Without SOC included, the calculated HSE bandgap is 3.13 eV with the parameters above. The SOC splits the double degenerate VBM and the gap is reduced by 0.37 eV. We find that the change of VBM contributes the most to the change in the value of band gap upon turning SOC. The VBM shifts upward about 0.32 eV while the CBM shifts downward about 0.05 eV.

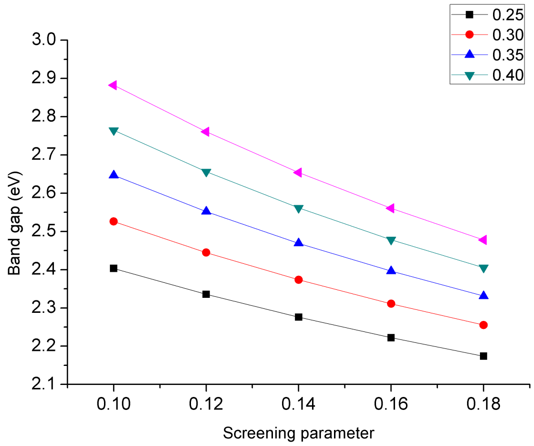

Supplementary Figure 1. HSE SOC calculation for the band gap as a function of the parameters of HSE functionals. The experimental optical band gap is about $1.85 - 1.99$ eV [9, 10], however, to reproduce the fundamental band gap (~ 2.7 eV) due to the large exciton energy, the mixing and screening parameters need to be modified.

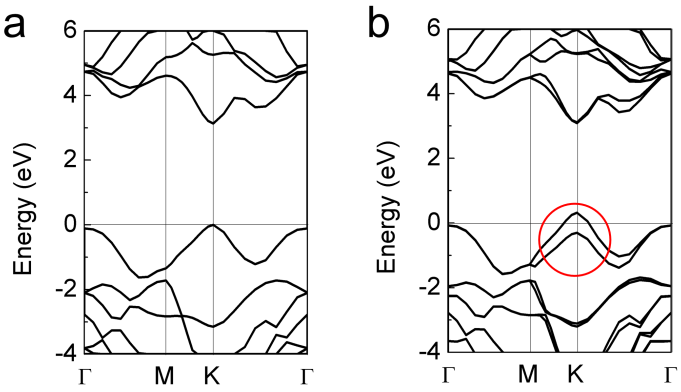

Supplementary Figure 2. The calculated HSE band gap of primitive WS_2 (a) without and (b) with SOC included. The black dash line indicates the energy referenced to the VBM without SOC included. We aligned the energy level in (b) to see the relative shift caused by SOC. The SOC effect shrinks the gap to 2.76 eV and splits the VBM by about 0.61 eV as denoted by red circle.

SUPPLEMENTARY NOTE 2: SUBSTRATE EFFECT

During experiment, the Fermi level of WS_2 is aligned by the graphene substrate so it is crucial to determine the influence of substrate on the electronic structure of WS_2. We use 5×5 supercell of graphene and 4×4 supercell of WS_2. The graphene layer is stratched to match WS_2, corresponding to 1% strain on graphene which is tolerable. The band structures with and without graphene substrate are shown in Supplementary Figure 3. The defect bands lie at the same position with and without the graphene substrate included.

Beside the graphene substrate, we also consider hexagonal boron nitride (hBN) as substrate and capping layer since it could protect the C_S defect and avoid hydrogen reattachment. Similar to graphene substrate, we use 5×5 supercell of hBN layers on top and bottom and 4×4 supercell of WS_2 as a middle layer. With the hBN included, the heterostructure exhibits type-I band alignment, as shown in Supplementary Figure 4. The defect level of the neutral charge state only shifts by 0.06 eV. With larger models, the energy difference can reduce to 0.01 eV. We demonstrate this by applying 8×8 supercell of WS_2 and 8×8 supercell of hBN sandwich layer structure. In this case, the geometry optimization was achieved within the affordable PBE functional and the electronic structure was calculated by HSE at that geometry as a single self-consistent calculation. We conclude that the weak van der Waals interaction between the layers minutely influences the electronic structure of WS_2, therefore hBN would be an excellent protective layer.

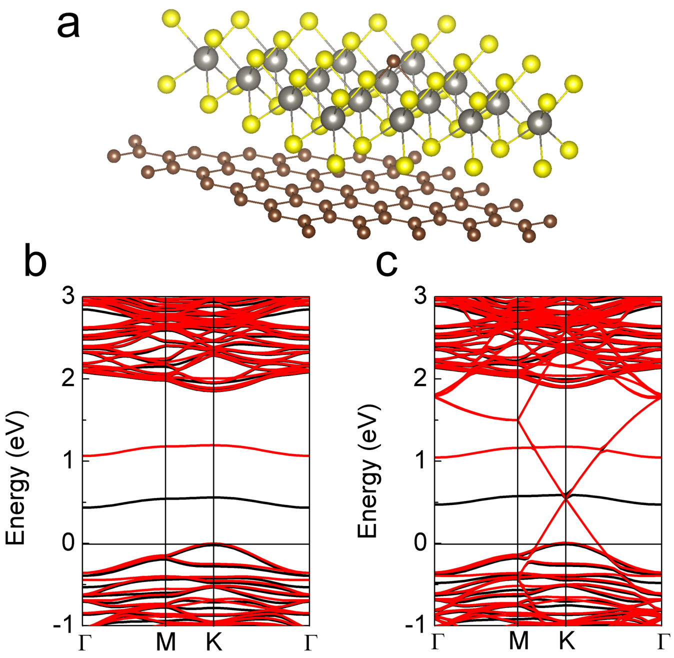

Supplementary Figure 3. (a) Graphene substrate (below) and WS_2 monolyer (top) heterostructure. The simulated PBE band structure of (b) freestanding WS_2 layer with C_S^- is almost unchanged compared with (c) the bilayer model. The black and red lines indicate the spin up and spin down channels. Two flat bands represent the defect bands that arise due to the relatively small supercell and interaction of defect wave functions with their periodic images. The VBM of WS_2 is referenced to zero.

SUPPLEMENTARY NOTE 3: CHARGE CORRECTION

It is well known that, in DFT simulation, the charged system with finite size supercell technique suffers from artificial electrostatic interaction between the periodic defect images and background charge. Therefore, correction scheme is needed to remove this interaction from both the total energy calculation and Kohn-Sham (KS) defect levels. The relative stability of charged defect could be evaluated by the formation energy. Here for the C_S in WS_2 it is given by

$$E_f^q = E_d^q - E_{\text{perfect}} + \mu_c - \mu_s + q\left(\epsilon_{\text{VBM}}^{\text{perfect}} + \epsilon_{\text{Fermi}}\right) + E_{\text{corr}}(q)\,, \tag{1}$$

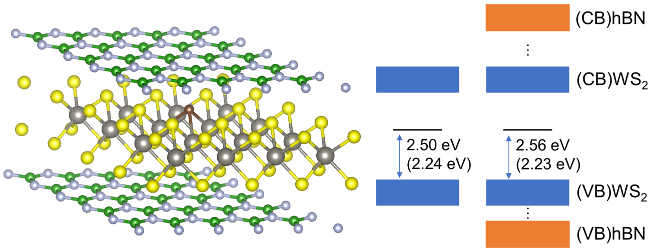

Supplementary Figure 4. The WS_2 with hBN substrate and capping layer and the band diagram. The solid lines indicate the defect level within the band gap of the pristine WS_2 (left) and WS_2 with hBN (right). The numbers in parentheses are calculated within a larger supercell model.

where E_d^q is the total energy of the system with defect at q charge state and E_{perfect} is the total energy of the pristine system without defect. The μ_c and μ_s are the chemical potential of carbon and sulfur atoms, respectively, and can be derived from their pure bulk structure. The Fermi level ϵ_{Fermi} represents the chemical potential of electron reservoir and should be aligned to the VBM energy of perfect WS_2, $\epsilon_{\text{VBM}}^{\text{perfect}}$. The $E_{\text{corr}}(q)$ is the correction term for the charged system due to the existence of electrostatic interactions with periodic boundary condition. This could be accomplished through supercell size scaling method and Freysoldt-Neugebauer-Van der Walle (FNV) correction as mentioned in the main text. [11] The FNV corrected formation energy demonstrates excellent agreement with the extrapolated value at infinite supercell size.

As a benchmark, we first perform the PBE calculation on various supercell sizes and all the atoms are fully relaxed. The models considered here range from 4×4 to 11×11. We fit the formation energy with a polynomial form as

$$E_f(a) = A + \frac{B}{a} + \frac{C}{a^2} + \frac{D}{a^3}, \tag{2}$$

where A corresponds to the isolated model energy. The fitted parameters are $A = 4.707$, $B = -20.686$, $C = 277.421$ and $D = -1099.563$. Then the FNV charge correction scheme is employed and the corrected formation energy agrees well with the extrapolated one, as shown in Supplementary Figure 5a. For the specified 6×6 supercell we use in the manuscript, we further correct the HSE formation energy as denoted by triangle. The correction energy is 0.448 eV which is very close to 0.486 eV with PBE functionals, manifesting the independence of charge correction of functionals used. The conclusion is still valid for excited states as listed in Supplementary Table 1. We also did correction based on recently proposed self-consistent potential correction (SCPC) method, the correction energy is 0.42 eV and confirms the validation of our correction.

Without charge correction, the HSE calculated occupied state is 0.51 eV above VBM while the unoccupied state is above the conduction band minimum (CBM). Supplementary Figure 5b shows the correction for defect KS levels with supercell size scaling method. For the negative charge state, both the occupied and unoccupied a_1 defect levels shift upward. The KS eigenvalue correction can be calculated with [12, 13]:

$$E_{corr}^{KS} = -\frac{2}{q} E_{corr}^{FNV}. \tag{3}$$

According to data in Supplementary Table 1, the correction energy is roughly 0.9 eV for both PBE and HSE functionals. With PBE functionals, the occupied state locates at 1.608 eV above VBM while the unoccupied state locates at 2.245 eV above VBM. Clearly the unoccupied state is already elevated above the CBM, which turns out that the observed unoccupied state does not belong to C_S^-. This is consistent with HSE result that the occupied state shifts to 1.406 eV above VBM while the unoccupied state resides at 4 eV above VBM. For the degenerate e state, the shift of defect levels is negligible.

Supplementary Table 1. FNV correction energy ($E_{\text{corr}}^{\text{FNV}}$) for 6×6 model at ground state (GS) and excited state (EX).

Configuration	PBE GS	HSE GS	HSE EX1	HSE EX2
Energy (eV)	0.486	0.448	0.451	0.442

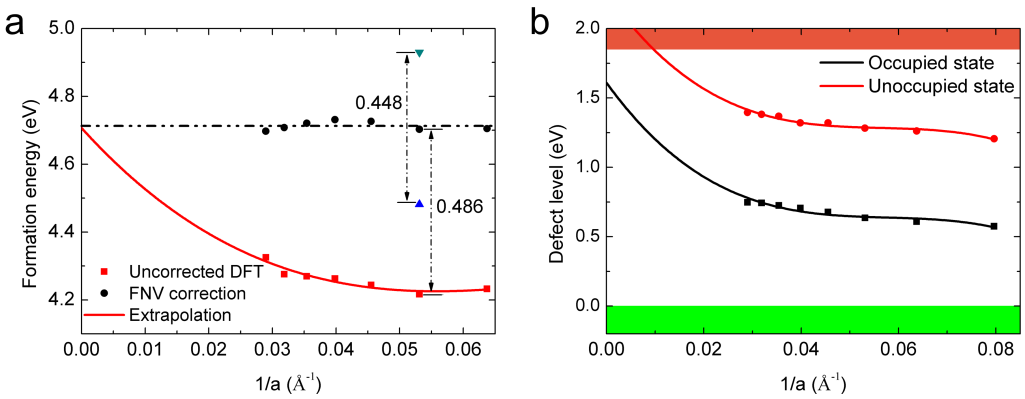

Supplementary Figure 5. Charge correction for formation energy and defect levels. (a) Red square indicates the uncorrected formation energy with different supercell sized of DFT PBE result. The fitted curve is shown by red line and extrapolated to infinite size. Black dot indicates the FNV corrected PBE formation energy and the black dash line represents the averaged FNV formation energy. The blue triangle is the HSE formation energy and green triangle is the FNV corrected result. (b) The black square and red dot represent the PBE calculated occupied and unoccupied defect KS levels. The black and red lines are fitting result and extrapolate to infinite size. The green and orange areas denote the VB and CB.

SUPPLEMENTARY NOTE 4: EXICTED STATES OF C_S^-

For the first excitation (EX1), in spin up channel the electron in the defect level could be excited to the CBM. The zero-phonon-line (ZPL) is calculated to evaluate the energy of excitation. Supplementary Figure 6 depicts the energy level diagram. Two a_1 levels share similar energy while the originally degenerate CBM splits. The energy difference between occupied and unoccupied levels is 0.623 eV. The calculated ZPL is 1.457 eV as listed in Supplementary Table 2.

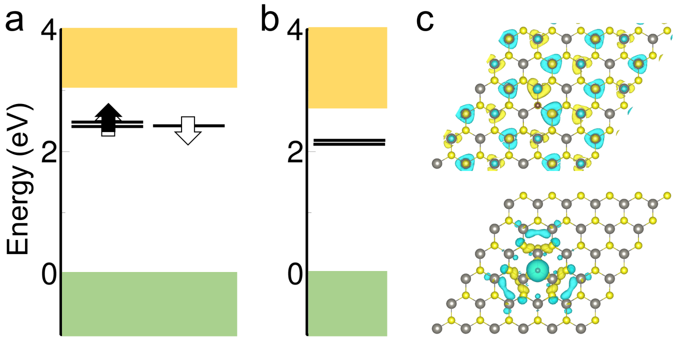

Supplementary Figure 6. Electronic structure of the first excited state of C_S^-. The energy levels diagram of C_S^- at first excited state without SOC (a) and with (b) SOC effect. The unfilled arrows indicate unoccupied states. (c) Spatial wave function of the unoccupied defect levels and occupied CBM in spin up channel. The isosurface is set to 0.00068 Å^3.

It is shown in Supplementary Figure 7 that the second excited state (EX2) is much more complicated than EX1 is. Despite there is no occupied defect level in gap in the ground state electronic configuration, the unoccupied e level appears in the gap upon filling the state by photoexcitation which constitutes of a defect-to-defect transition. This could be due to the strong interaction between the defect states since both the e and a_1 states contain large d orbital components of tungsten atoms.

SUPPLEMENTARY NOTE 5: SOLUTION FOR THE JTE AND SOC

Previous studies on nitrogen-vacancy (NV) center in diamond pointed out that the ^{3}E excited state is unstable in C_{3v} symmetry due to the Jahn-Teller effect (JTE). [14–16] The JTE could reduce or even quench the SOC with damped factor p. This JTE is known as $E \otimes e$ dynamic JT system which was investigated by Ham and Bersuker. [17, 18]

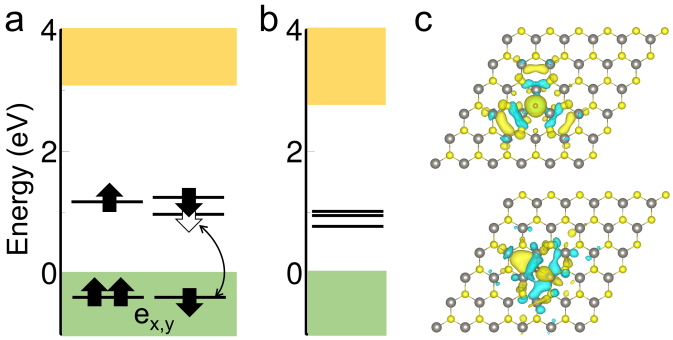

Supplementary Figure 7. Electronic structure of the second excited state of C_S^-. The energy levels diagram of C_S^- at first excited state without SOC (a) and with (b) SOC effect. The unfilled arrows indicate unoccupied states. (c) Spatial wave function of the defect levels in spin down channel. The isosurface is set to 0.00068 $Å^3$.

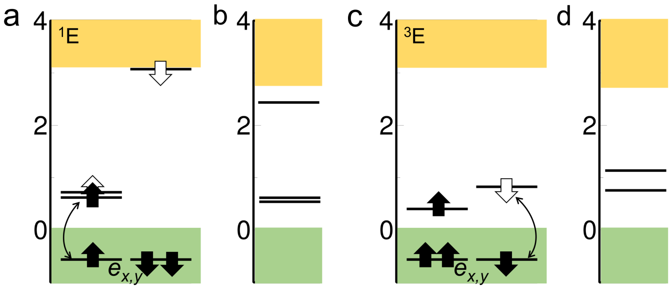

Supplementary Figure 8. Electronic structure of the excited state of C_S^0. The energy levels diagram of C_S^0 at ^{1}E excited state (a) without SOC and (b) with SOC effect. The energy levels diagram of C_S^0 at ^{3}E excited state (c) without SOC and (d) with SOC effect. The unfilled arrows indicate unoccupied states.

The symmetry breaking e phonons or local vibration modes reduce the high symmetry configuration that may couple the double degenerate E electron wave functions.

The SOC can be regarded as perturbation term if the JT energy is orders magnitude higher than SOC effect. Then $\lambda_{Ham} = |\langle\widetilde{\Psi}_\pm|\hat{H}_{SOC}|\widetilde{\Psi}_\pm\rangle| = p\lambda$. $|\widetilde{\Psi}_\pm\rangle$ is the vibronic wavefuntions caused by JT distortion. However, our calculated JT energy E_{JT} from C_{3v} to C_s is 89.9 meV which is very close to SOC and neither of them could be treated as perturbation. Then the SOC and electron-phonon interaction couple with each other and should be simultaneously solved.

We first discuss the system from pure electronic point of view. $\mathcal{A}$ is the usual anti-symmetrization operator: $\mathcal{A}|xy\rangle = \frac{1}{\sqrt{2}}(|xy\rangle - |yx\rangle)$. So the four states in the main text could be expressed by:

$$|E_{12}\rangle = \begin{cases} \mathcal{A}|e_+^\uparrow a^\uparrow\rangle \\ \mathcal{A}|e_-^\downarrow a^\downarrow\rangle \end{cases}$$

$$|A_{12}\rangle = \begin{cases} \mathcal{A}|e_-^\uparrow a^\uparrow\rangle \\ \mathcal{A}|e_+^\downarrow a^\downarrow\rangle \end{cases}$$

Supplementary Table 2. The calculated ZPL energies for two charge states at C_{3v} symmetry.

		ZPL (eV)
C_S^-	EX1	1.457
C_S^-	EX2	1.803
C_S^0	Singlet	1.062
C_S^0	Triplet	0.958

$$|E_{xy}\rangle = \begin{cases} \left[\mathcal{A}|e_+^\uparrow a^\downarrow\rangle + \mathcal{A}|e_+^\downarrow a^\uparrow\rangle\right]/\sqrt{2} \\ \left[\mathcal{A}|e_-^\uparrow a^\downarrow\rangle + \mathcal{A}|e_-^\downarrow a^\uparrow\rangle\right]/\sqrt{2} \end{cases}$$

$$|^1E\rangle = \begin{cases} \left[\mathcal{A}|e_+^\uparrow a^\downarrow\rangle - \mathcal{A}|e_+^\downarrow a^\uparrow\rangle\right]/\sqrt{2} \\ \left[\mathcal{A}|e_-^\uparrow a^\downarrow\rangle - \mathcal{A}|e_-^\downarrow a^\uparrow\rangle\right]/\sqrt{2} \end{cases}$$

The single particle SOC operator is

$$H^{(1)} = \lambda_z^{(1)} \hat{l}_z \hat{s}_z.$$

The two particle SOC operator is the sum of the two single particle SOC operators,

$$H^{(2)} = H^{(1)} \otimes \hat{I} + \hat{I} \otimes H^{(1)} = \lambda_z^{(2)} \hat{L}_z \hat{S}_z.$$

$\mathcal{A}|e_\pm^\uparrow a^\downarrow\rangle$ and $\mathcal{A}|e_\pm^\downarrow a^\uparrow\rangle$ are eigenstates of $H^{(2)}$

$$\begin{aligned} H^{(2)}\mathcal{A}|e_\pm^\uparrow a^\downarrow\rangle &= H^{(2)}\left[|e_\pm^\uparrow a^\downarrow\rangle - |a^\downarrow e_\pm^\uparrow\rangle\right]/\sqrt{2} \\ &= \frac{1}{\sqrt{2}}\left[|\left(H^{(1)}e_\pm^\uparrow\right)a^\downarrow\rangle - |a^\downarrow\left(H^{(1)}e_\pm^\uparrow\right)\rangle\right] \\ = \frac{\lambda_z^{(1)}}{\sqrt{2}}&\left[\pm\frac{1}{2}|e_\pm^\uparrow a^\downarrow\rangle \mp \frac{1}{2}|a^\downarrow e_\pm^\uparrow\rangle\right] = \pm\frac{\lambda_z^{(1)}}{2}\mathcal{A}|e_\pm^\uparrow a^\downarrow\rangle. \end{aligned}$$

Note that if there is no triplet-singlet splitting, $|E_{xy}\rangle$ and $|^1E\rangle$ would be degenerate. And if you switch on the SOC, it would lift the degeneracy, but the eigenstates would be still single determinant configurations.

So the total Hamiltonian is

$$\begin{aligned} \hat{H} = \hat{W}_{\text{ee}} + H^{(2)} &= 0|E_{xy}\rangle\langle E_{xy}| + \Lambda|^1E\rangle\langle^1E| + \\ \frac{\lambda_z^{(1)}}{2}&\left(\mathcal{A}|e_+^\uparrow a^\downarrow\rangle\langle e_+^\uparrow a^\downarrow|\mathcal{A} + \mathcal{A}|e_-^\downarrow a^\uparrow\rangle\langle e_-^\downarrow a^\uparrow|\mathcal{A}\right. \\ &\left.-\mathcal{A}|e_-^\uparrow a^\downarrow\rangle\langle e_-^\uparrow a^\downarrow|\mathcal{A} - \mathcal{A}|e_+^\downarrow a^\uparrow\rangle\langle e_+^\downarrow a^\uparrow|\mathcal{A}\right) \end{aligned}$$

Therefore, the 8×8 matrix that describes the electronic degrees of freedom is the following,

$$\begin{pmatrix} -\lambda &&&&&&& \\ & -\lambda &&&&&& \\ && 0 &&&& \lambda & \\ &&& 0 &&&& \lambda \\ &&&& +\lambda &&& \\ &&&&& +\lambda && \\ && \lambda &&&& \Delta & \\ &&& \lambda &&&& \Delta \end{pmatrix} \begin{matrix} \mathcal{A}|e_+^\uparrow a^\uparrow\rangle \\ \mathcal{A}|e_-^\downarrow a^\downarrow\rangle \\ \left[\mathcal{A}|e_+^\uparrow a^\downarrow\rangle + \mathcal{A}|e_+^\downarrow a^\uparrow\rangle\right]/\sqrt{2} \\ \left[\mathcal{A}|e_-^\uparrow a^\downarrow\rangle + \mathcal{A}|e_-^\downarrow a^\uparrow\rangle\right]/\sqrt{2} \\ \mathcal{A}|e_-^\uparrow a^\uparrow\rangle \\ \mathcal{A}|e_+^\downarrow a^\downarrow\rangle \\ \left[\mathcal{A}|e_+^\uparrow a^\downarrow\rangle - \mathcal{A}|e_+^\downarrow a^\uparrow\rangle\right]/\sqrt{2} \\ \left[\mathcal{A}|e_-^\uparrow a^\downarrow\rangle - \mathcal{A}|e_-^\downarrow a^\uparrow\rangle\right]/\sqrt{2} \end{matrix}$$

In our present approximation, the Jahn-Teller effect originates in the real e_x/e_y character instead of the complex $e_\pm = (e_x \pm ie_y)/\sqrt{2}$ that is important towards the spin-orbit coupling.

$$\hat{H}_{\text{JT}}^{(1)} = \hbar\omega(a_X^\dagger a_X + a_Y^\dagger a_Y + 1) + F\left(\hat{\sigma}_z\hat{X} + \hat{\sigma}_x\hat{Y}\right) + G\ldots,$$

where

$$\hat{\sigma}_z = |e_x\rangle\langle e_x| - |e_y\rangle\langle e_y| \qquad \hat{\sigma}_x = |e_x\rangle\langle e_y| + |e_y\rangle\langle e_x|.$$

We need to transform it to two-particle operator formalism as

$$\hat{H}^{(2)}_{\mathrm{JT}} = \hat{H}^{(1)}_{\mathrm{JT}} \otimes \hat{I} + \hat{I} \otimes \hat{H}^{(1)}_{\mathrm{JT}}.$$

Additionally, the Jahn-Teller interaction cannot connect the $|E_{12}\rangle, |A_{12}\rangle$ with $|E_{xy}\rangle, |^1E\rangle$ subspace. That is, all $|E_{12}\rangle, |A_{12}\rangle$'s are maximal in spin, $\uparrow\uparrow$ or $\downarrow\downarrow$ ($m_S = \pm 1$), but the singlets and $m_S = 0$ states constitutes of antiparallel spins, $\uparrow\downarrow$ or $\downarrow\uparrow$. And since the Jahn-Teller operator cannot flip the spin there are no offdiagonal matrix elements appear between them. Also, the spin-orbit does not induce mixing, thus the $m_S = \pm 1$ and $m_S = 0$ subspace can be described fully independently. The two-particle Pauli matrices for Jahn-Teller effect acting inside the $|E_{12}\rangle, |A_{12}\rangle$ states are

$$\hat{\sigma}_z = \mathcal{A}|e_x^{\uparrow}a^{\uparrow}\rangle\langle e_x^{\uparrow}a^{\uparrow}|\mathcal{A} - \mathcal{A}|e_y^{\uparrow}a^{\uparrow}\rangle\langle e_y^{\uparrow}a^{\uparrow}|\mathcal{A} + \mathcal{A}|e_x^{\downarrow}a^{\downarrow}\rangle\langle e_x^{\downarrow}a^{\downarrow}|\mathcal{A} \\ - \mathcal{A}|e_y^{\downarrow}a^{\downarrow}\rangle\langle e_y^{\downarrow}a^{\downarrow}|\mathcal{A}$$

$$\hat{\sigma}_x = \mathcal{A}|e_x^{\uparrow}a^{\uparrow}\rangle\langle e_y^{\uparrow}a^{\uparrow}|\mathcal{A} + \mathcal{A}|e_y^{\uparrow}a^{\uparrow}\rangle\langle e_x^{\uparrow}a^{\uparrow}|\mathcal{A} + \mathcal{A}|e_x^{\downarrow}a^{\downarrow}\rangle\langle e_y^{\downarrow}a^{\downarrow}|\mathcal{A} \\ + \mathcal{A}|e_y^{\downarrow}a^{\downarrow}\rangle\langle e_x^{\downarrow}a^{\downarrow}|\mathcal{A}$$

After considering vibronic coupling with the additional Jahn-Teller effect, the SOC spliting between $|E_{12}\rangle$ and $|A_{12}\rangle$ levels will be reduced to $p\lambda$.

$$\begin{pmatrix} -p\lambda & & & \\ & -p\lambda & & \\ & & +p\lambda & \\ & & & +p\lambda \end{pmatrix} \begin{matrix} |\widetilde{E}_{12}\rangle \\ \\ |\widetilde{A}_{12}\rangle \\ \end{matrix}$$

For $\hat{H}_{\mathrm{JT}}$, we solve it using series expansion

$$|\widetilde{\Psi}_{\pm}\rangle = \sum_{nm} \left[c_{nm}|E_{\pm}\rangle \otimes |n,m\rangle + d_{nm}|E_{\mp}\rangle \otimes |n,m\rangle\right], \; n,m = 0,1,2,\ldots \tag{4}$$

where c_{nm} and d_{nm} are quantum oscillator coefficients. p could be calculated from $p = \sum_{nm}\left[c_{nm}^2 - d_{nm}^2\right]$ which is 0.017 indicating a giant reduction or almost quenched SOC due to JTE. Ham also numerically proposed fitting function $p = \exp\left(-1.974 \times \chi^{0.761}\right)$, where $\chi = E_{\mathrm{JT}}/\hbar\omega_e$. Here $\chi = 2.99$ and should be close to Huang-Rhys (HR) factor S between C_{3v} to C_s configuration (2.55). Therefore, the deduced $p = 0.011$ from this approximation is close to our calculation with using the DJT Hamiltonian.

SUPPLEMENTARY NOTE 6: PHONON PROPERTIES OF C_S^-

Based on Franck-Condon approximation, the phonon vibration could be evaluated through the overlap of phonon mode between electronic ground state and excited state. Huang-Rhys (HR) approximation further simplifies the mechanism that the interaction is equivalent between the defect and the lattice during excitation meaning the phonons are the same in the electronic ground and excited states.

The calculated HR factors are listed in Supplementary Table 3. We predict the Debye-Waller factor as e^{-S}. With PBE functionals, the calculated HR factor for the first and second excited states are 3.00 and 4.82 in C_{3v} symmetry, respectively. These values agree well with previous findings. [19] We notice that the HSE functionals yield larger HR factors which might be attributed to the phonon calculation using the PBE functionals in the procedure.

The calculated phonon sideband of the PL spectrum of the two optical transitions are plotted in Supplementary Figure 10. The partial HR factors S_k represents the average number of phonons that emitted during excitation for specified phonon mode as illustrated in Supplementary Figure 11. For excitation 1 (EX1) with C_{3v} symmetry, we

could screen out dominant localized phonon modes at 23 meV and 77 meV contributing to the neighbour sulphur atom. The 23-meV mode tends to shift the first neighbouring tungsten atoms towards the carbon impurity and then stretch the perpendicular distance between the carbon and underneath sulfur atoms, as shown in Supplementary Figure 12. The 77-meV mode is an out-of-plane vibration of carbon solely. We also observe there are two delocalized modes at 14 meV and 18 meV. This might be attributed to the involvement of the band edge states during excitation. In the second excitation, the 23-meV mode still dominate while the 77-meV mode disappears because the carbon atom is relaxed out-of-plane during this excitation. One more delocalized mode appears at 51 meV with PBE level but not observable at HSE level.

Supplementary Table 3. Calculated Huang-Rhys (HR) factors S and Debye-Waller factor (DWF) with different configurations at C_{3v} symmetry.

Configuration	S(PBE)	S(HSE)	DWF(PBE)	DWF(HSE)
EX1	3.00	10.35	0.050	0.00003
EX2	4.82	5.08	0.008	0.0062
Singlet	3.00	5.08	0.05	0.0062
Triplet	3.16	6.28	0.043	0.0018

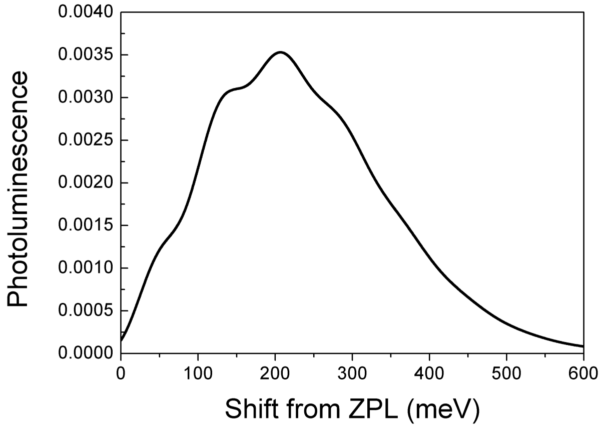

Supplementary Figure 9. The simulated photoluminescence of C_S^0.

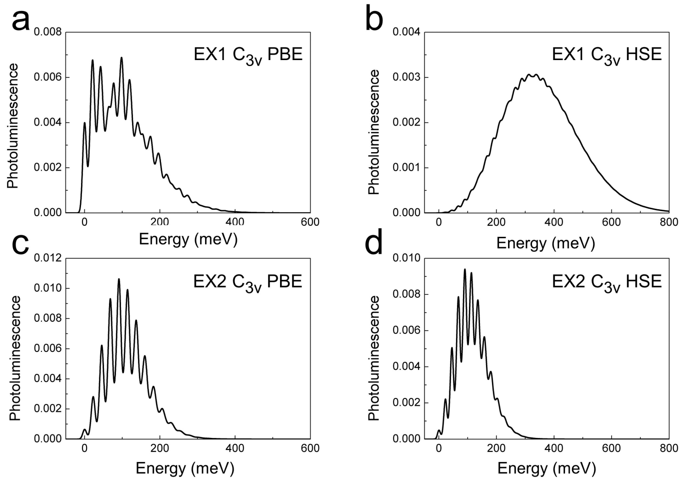

Supplementary Figure 10. The simulated photoluminescence of C_S^-.

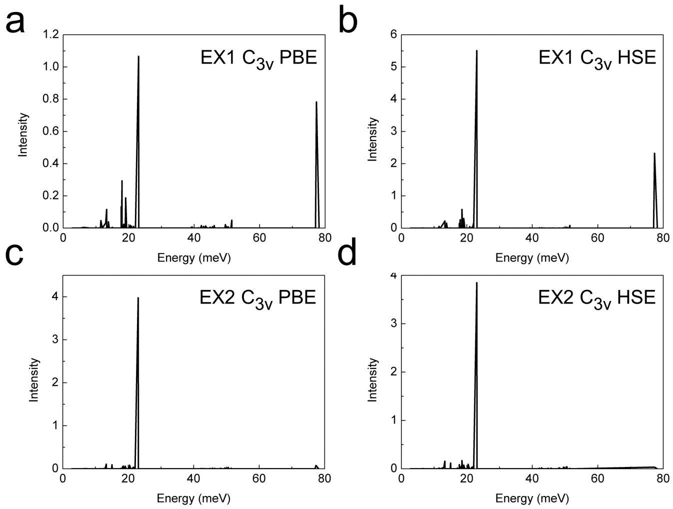

Supplementary Figure 11. The partial Huang-Phys factors of C_S^-.

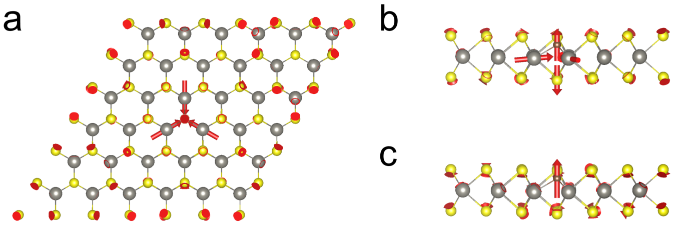

Supplementary Figure 12. Localized phonon modes. (a) top and (b) side view of phonon mode at 23 meV. (c) Side view of phonon mode at 78 meV.

SUPPLEMENTARY NOTE 7: HYPERFINE CONSTANT CALCULATION

The calculated hyperfine constants are shown in Supplementary Table 4. The electron spin coherence time (T_2) is particlular important for quantum applications and generally would be effected by proximate nuclear spins around the defect in the host material. In Supplementary Figure 13 we depict the distribution of the hyperfine constants of nuclear spins around the central carbon atom. The coherence time in WS_2 is about 11 ms therefore the farther tungsten atoms colored with purple could be used to store quantum information which do not significantly shorten the coherence time of the defect electron spin.

Supplementary Table 4. The calculated hyperfine constant with HSE functionals with contribution of the spin polarization of the core states (A_{1c}) in the Fermi-contact term. The first neighbouring W atoms and the S atom below C_S defect are considered. The natural isotopic abundance of ^{183}W ($I = 1/2$), ^{33}S ($I = 3/2$), and ^{13}C ($I = 1/2$) are 14.31%, 0.76%, and 1.07% respectively. The subnotations of W indicate the first, second and third nearest atoms. The average of the principal values of the hyperfine tensor (Average) is defined as $(A_{xx} + A_{yy} + A_{zz})/3$.

Atom	A_{xx} (MHz)	A_{yy} (MHz)	A_{zz} (MHz)	Average (MHz)
$^{183}W_1$	5.24	-0.35	10.53	5.14
$^{183}W_2$	-3.32	-4.35	0.70	-2.32
$^{183}W_3$	-0.73	-1.15	2.00	0.04
^{33}S	1.57	1.57	10.56	4.57
^{13}C	111.39	111.39	190.47	137.75

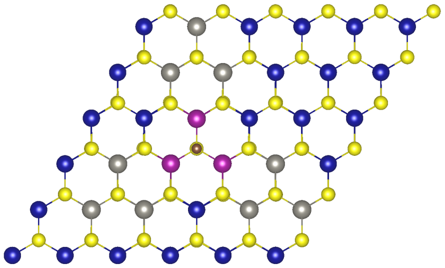

Supplementary Figure 13. The hyperfine constants of tungsten atoms. The purple ones with hyperfine constants around 10 MHz and the blue ones indicate hyperfine constants less than 1 MHz.

SUPPLEMENTARY NOTE 8: DISCUSSION ON THE NATURE OF THE EXCITED STATE OF THE NEUTRAL C DEFECT

We assumed that the lowest energy excited state of the neutral C defect can be described as promoting an electron from the e orbital to the empty a_1 orbital in the gap. We calculated the excited state and the energy by ΔSCF method. The choice of the selection of the occupied and unoccupied (virtual) orbitals is based on the insight of the possible excitation routes but, in principle, it might be too restrictive and several pairs of occupied and virtual orbitals might be incorporated to describe the exciton wavefunction as the occupied orbital resides resonant in the valence band in the ground state electronic configuration.

We carried out further ΔSCF and many-body perturbation theory GW+BSE calculations [20, 21] within Γ-point approximation. We here use a smaller supercell, 4×4, in order to reach close-to-converged calculation for the GW method which is very computationally demanding in the VASP implementation as more than 1500 bands were included in the single-shot G_0W_0 calculation. The energy cutoff for the response function is set to be 150 eV. The Tamm-Dancoff approximation was used to solve BSE. The highest seven valence bands and seven lowest conduction bands are considered as basis for the excitonic state in the BSE procedure. We found that the results do not change by including more valence bands in this procedure. The calculations are based on the optimized HSE functional which resulted in the optimized geometry and the electronic structure of the neutral carbon defect in WS_2. Here, we do not consider spin-orbit interaction. The ΔSCF procedure is applied at fixed geometry as obtained in the ground state, so vertical excitation energy is calculated. Although, this procedure is not entirely converged because of the small supercell but provides qualitatively good BSE results.

We find that the empty a1 defect level in the band gap by HSE agrees within 0.07 eV with G_0W_0 quasi-particle level with respect to the calculated valence band maximum (see Supplementary Figure 14a). The ΔSCF method yields 1.43 eV vertical excitation energy in this supercell. The hole wavefunction is localized on the $e_{x,y}$ orbitals and the hole level pops up in the band gap, similarly to that in the large 6×6 supercell calculation. In the BSE absorption spectrum the first peak has a significant oscillator strength, and the weight of $e_{x,y}$ orbitals in the exciton state is about 86%. The dominant contribution of these orbitals in building up the hole part of the exciton justifies our procedure in ΔSCF. On the other hand, the first peak in the BSE absorption spectrum occurs at 1.77 eV (see Supplementary Figure 14d). The source of difference in the calculated ΔSCF and BSE excitation energies can be either the possibly missing 14% electron-hole pairs in building up the exciton in the ΔSCF procedure (with reducing the weight of the $e_{x,y}$ orbitals to 86%) or the missing back action on the quasi-particle wavefunction of the BSE method within Tamm-Dancoff approach as explained below.

According to our previous findings in other materials with similar electronic structure (defects in diamond, silicon carbide and silicon), the localization of the hole is highly critical which would decrease the excitation energy. This is analyzed by the inverse participation ratio method for the e and a_1 orbitals. The corresponding orbitals are plotted in Supplementary Figure 14b. One can see that the empty e_x orbital in the ΔSCF orbital is more localized than the e_x orbital in the ground state electronic configuration in the 4×4 calculation, and the empty e_x level pops up in the gap in the ΔSCF procedure. We find that the e_x orbital significantly localized when emptied whereas the a_1 orbital becomes less localized when occupied. As a consequence, the overlap between the e_x and a_1 electron densities increases by ΔSCF with respect to that in the ground state DFT. This should result in a stronger attractive interaction between these particles with lowering its total energy. Obviously, the localization of hole part of the exciton wavefunction cannot occur by BSE method within Tamm-Dancoff approach (see Fig. 14b). We note that the localization of the e_x orbital changes by +70% whereas it changes for a_1 orbital by −17% in the 6×6 supercell.

In summary, we conclude that the BSE method supports the dominant contribution of the selected orbitals in the ΔSCF method and the significant oscillator strength (optically allowed transition).

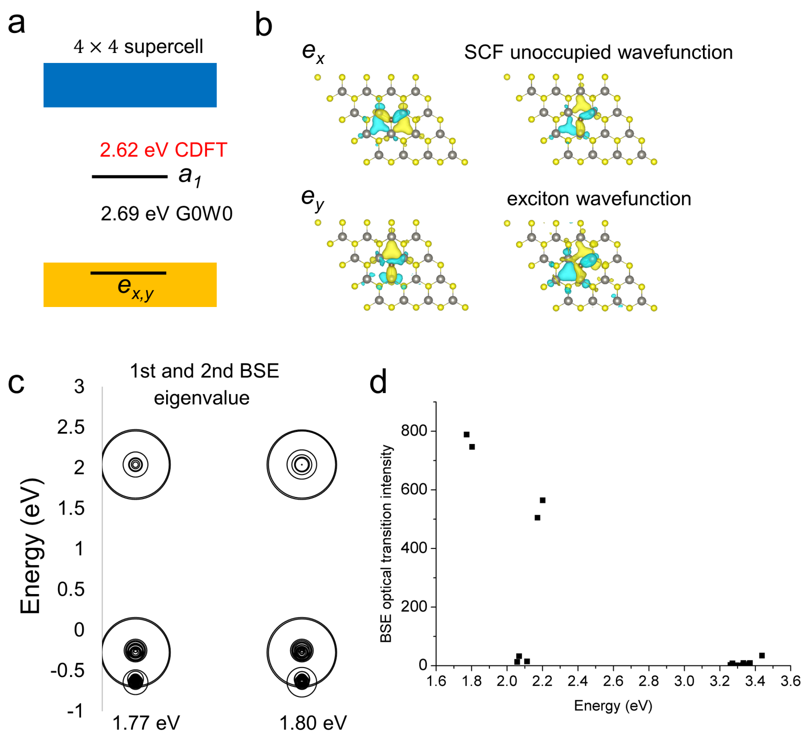

Supplementary Figure 14. GW+BSE calculation for 4 × 4 WS_2 supercell. (a) G_0W_0 band diagram of neutral carbon defect in WS_2 without spin-orbit correction. Numbers with red color indicate the calculated energy with HSE functionals. (b) The e_x and e_y wavefunctions are obtained by HSE wavefunctions in the ground state electron configuration which are fully occupied as shown in (a). The "SCF unoccupied wavefunction" plots the unoccupied e orbital in the ΔSCF calculation, whereas the "exciton wavefunction" is the hole part of the exciton wavefunction of the lowest excitation energy which is built upon the linear combination of states close to the valence band maximum based on the BSE wavefunction coefficients. The isosurface is set to 0.002 $e/\AA^3$. (c) The BSE fat band for the first and second BSE eigenstates at Γ point. The e and a_1 hole-electron pair contributes the most to BSE exciton eigenstate. (d) The optical transition intensity calculated within BSE. The first two peaks correspond to the e to a_1 transition.

SUPPLEMENTARY NOTE 9: NON-RADIATIVE TRANSITION RATE

The non-radiative transition rate between two electronic configurations under perturbation can be calculated with

$$\Gamma_{non} = \frac{2\pi}{\hbar} g W_{if}^2 X_{if}(T),$$
$$X_{if}(T) = \sum_{n,m} p_{in} |\chi_i|\hat{Q} - Q_0|\chi_f|^2 \times \delta(m\hbar\omega_i - m\hbar\omega_f + \Delta E_{if}),$$
$$W_{if} = \langle\psi_i|\partial_Q \hat{H}|\psi_f\rangle.$$

Here, W_{if} is the electronic term and $X_{if}(T)$ is temperature dependent phonon term. g is the equivalent energy-degenerate atomic configurations and p_{in} is the thermal population of the initial state (i), and f corresponds to the final state. The phonon matrix $|\chi_i|\hat{Q} - Q_0|\chi_f|$ sums up the harmonic oscillator wave functions that enter the non-radiative recombination process. ΔE_{if} is the calculated ZPL energy as described in the main text and ψ is the KS DFT wave function. Here two non-radiative processes are considered, one from ^1E singlet to ^3E triplet and the other one is from ^3E triplet to the singlet ground state, and the corrresponding W_{if} are 0.017 and 0.001 eVamu$^{-1/2}$Å^{-1}, respectively. We note that direct non-radiative process is possible (without any spin-flip) between ^3E and the singlet states because of the 2.1% singlet character in ^3E state. The dominant triplet character in ^3E may interact non-radiatively with the singlet states via spin-flip called intersystem crossing (ISC). The calculation method of the ISC rate is given in the main text. Then the ISC and non-radiative rates are calculated and shown in Supplementary Figure 15. The ^1E singlet to ^3E triplet transition is very fast due to the small energy splitting and HR factor (0.12).

The total transition rate from singlet to triplet is

$$\frac{1}{\tau} = \frac{1}{p_1 \tau_{\rm non}} + \frac{1}{p_3 \tau_{\rm ISC}}.$$

p_1 and p_3 are the fraction of singlet and triplet characters in the ^3E triplet state (triplet character is dominant, $p_1 \ll p_3$), respectively. The calculated lifetime of ISC is 0.68 ps and the direct non-radiative lifetime is about 38.2 ps (here, $g = 3$). The averaged lifetime is close to the ISC one going from ^1E to ^3E since it dominates the decay process. The non-zero spin sublevels $^3A_1$ could be linked with singlets ground state with the non-axial part $\lambda_{x,y}$ of SOC. We find that either ISC or direct non-radiative decay process between the triplet ^3E and $^1A_1$ ground state is relatively slow at low temperatures because of the large HR factor which greatly suppresses the $|\chi_i|\hat{Q} - Q_0|\chi_f|$ term. Thus we conclude that the radiative decay dominates for ^3E state at low temperatures and the lifetime is still sufficiently long to carry out quantum information operations. At elevated temperatures, the ISC rate starts to dominate over the radiative rate (about 6.8 MHz, see main text) which may be disadvantageous for qubit operations.

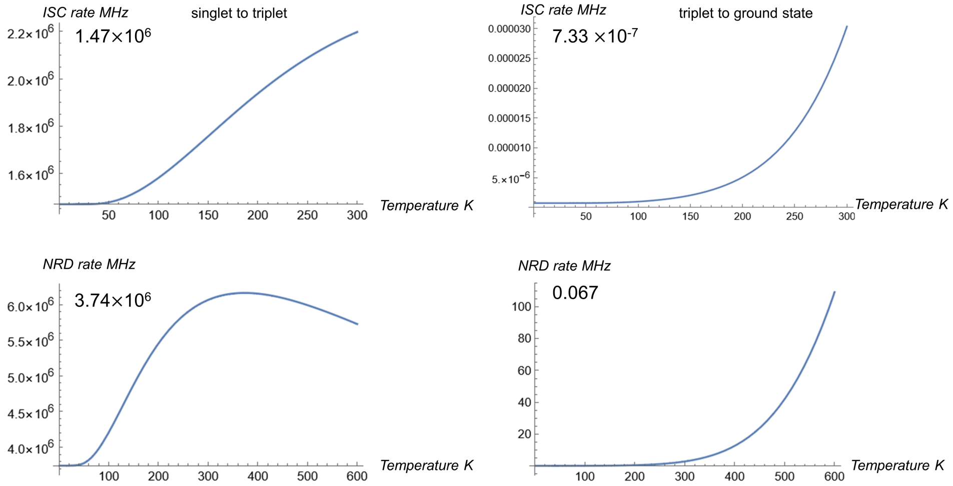

Supplementary Figure 15. The intersystem crossing (ISC) and non-radiative (NRD) rates as a function of temperature for ^1E singlet to ^3E triplet (singlet to trplet) and ^3E triplet to $^1A_1$ ground state (triplet to ground state). The values at zero kelvin temperature are explicitly given.

SUPPLEMENTARY REFERENCES

[1] He, K. *et al.* Tightly bound excitons in monolayer WSe_2. *Physical review letters* **113**, 026803 (2014).

[2] Chernikov, A. *et al.* Exciton binding energy and nonhydrogenic rydberg series in monolayer WS_2. *Physical review letters* **113**, 076802 (2014).

[3] Zhu, B., Chen, X. & Cui, X. Exciton binding energy of monolayer WS_2. *Scientific reports* **5**, 1–5 (2015).

[4] Ramasubramaniam, A. Large excitonic effects in monolayers of molybdenum and tungsten dichalcogenides. *Physical Review B* **86**, 115409 (2012).

[5] Shi, H., Pan, H., Zhang, Y.-W. & Yakobson, B. I. Quasiparticle band structures and optical properties of strained monolayer MoS_2 and WS_2. *Physical Review B* **87**, 155304 (2013).

[6] Drüppel, M. *et al.* Electronic excitations in transition metal dichalcogenide monolayers from an LDA+GdW approach. *Physical Review B* **98**, 155433 (2018).

[7] Ivády, V. *et al.* Theoretical unification of hybrid-DFT and DFT+U methods for the treatment of localized orbitals. *Physical Review B* **90**, 035146 (2014).

[8] Viñes, F., Lamiel-García, O., Chul Ko, K., Yong Lee, J. & Illas, F. Systematic study of the effect of HSE functional internal parameters on the electronic structure and band gap of a representative set of metal oxides. *Journal of computational chemistry* **38**, 781–789 (2017).

[9] Zhao, W. *et al.* Origin of indirect optical transitions in few-layer MoS_2, WS_2, and WSe_2. *Nano letters* **13**, 5627–5634 (2013).

[10] Zhang, F. *et al.* Carbon doping of WS_2 monolayers: Bandgap reduction and p-type doping transport. *Science advances* **5**, eaav5003 (2019).
[11] Freysoldt, C. & Neugebauer, J. First-principles calculations for charged defects at surfaces, interfaces, and two-dimensional materials in the presence of electric fields. *Physical Review B* **97**, 205425 (2018).
[12] Naik, M. H. & Jain, M. Coffee: Corrections for formation energy and eigenvalues for charged defect simulations. *Computer Physics Communications* **226**, 114–126 (2018). URL https://www.sciencedirect.com/science/article/pii/S0010465518300158.
[13] Chen, W. & Pasquarello, A. First-principles determination of defect energy levels through hybrid density functionals andGW. *Journal of Physics: Condensed Matter* **27**, 133202 (2015). URL https://doi.org/10.1088/0953-8984/27/13/133202.
[14] Thiering, G. & Gali, A. Ab initio magneto-optical spectrum of group-IV vacancy color centers in diamond. *Physical Review X* **8**, 021063 (2018).
[15] Thiering, G. & Gali, A. Ab initio calculation of spin-orbit coupling for an NV center in diamond exhibiting dynamic jahn-teller effect. *Physical Review B* **96**, 081115 (2017).
[16] Thiering, G. & Gali, A. The ($e_g \otimes e_u$)$\otimes$ Eg product Jahn–Teller effect in the neutral group-IV vacancy quantum bits in diamond. *npj Computational Materials* **5**, 1–6 (2019).
[17] Bersuker, I. *The Jahn-Teller Effect* (Cambridge University Press, 2006).
[18] Ham, F. S. Dynamical Jahn-Teller effect in paramagnetic resonance spectra: orbital reduction factors and partial quenching of spin-orbit interaction. *Physical Review* **138**, A1727 (1965).
[19] Cochrane, K. A. *et al.* Vibronic response of a spin-1/2 state from a carbon impurity in two-dimensional WS_2. *Nature Communications* **12**, 7287 (2021).
[20] Rohlfing, M. & Louie, S. G. Electron-hole excitations in semiconductors and insulators. *Physical review letters* **81**, 2312 (1998).
[21] Shishkin, M. & Kresse, G. Self-consistent GW calculations for semiconductors and insulators. *Physical Review B* **75**, 235102 (2007).
